# Self-Rated Emergency Core Nursing Competencies Among Emergency Nurses in Qassim, Saudi Arabia

**DOI:** 10.7759/cureus.32416

**Published:** 2022-12-11

**Authors:** Hadiya N AlRashedi, Bushra Alshammari, Maha AlOtaibi, Fawziha AlRashedi, Noor Alanazi, Effa AlOtaiby, Fatimah AlSayed

**Affiliations:** 1 Emergency Medicine, Qassim Health Cluster, Buraidah, SAU; 2 Nephrology, College of Nursing, University of Hail, Hail, SAU; 3 Surgery, Qassim Health Cluster, Buraidah, SAU; 4 Emergency Medicine, Diabetic Center, King Fahad Specialist Hospital, Buraidah, SAU; 5 Family Medicine, Albuser Primary Health Care Center, North Buraidah Health Sector, Albuser, SAU; 6 Surgery, Al Mredysia Primary Health Care Center, South Buraidah Health Sector, Buraidah, SAU; 7 Emergency Medicine, College of Nursing, University of Hail, Buraidah, SAU; 8 Family Medicine, Family Medicine Academy, Qassim Health Cluster, Buraidah, SAU

**Keywords:** saudi arabia, emergency department, nurses, emergency, core competencies

## Abstract

Introduction

Emergency situations require advanced and specialized knowledge and skills to handle urgent situations. However, there is a scarcity of literature on emergency nurses’ competencies. We assessed the competencies of nurses working in the emergency departments of hospitals in the Qassim region.

Methods

A cross-sectional study was conducted among 213 nursing staff at the emergency departments of all government hospitals in the Qassim region. A standardized tool was adopted for data collection which encompassed sociodemographic and self-reported competencies in nine emergency nursing domains, reflecting the core competencies using a 5-point Likert scale. Data was collected through an online survey. SPSS version 26 (IBM Corp., Armonk, USA) was used for data analysis. Linear regression analysis was carried out to explore the factors associated with competencies.

Results

A total of 213 nurses were included in the study with a mean age of 32.9±4.6 years. More than two-thirds of the nurses were female (69.5) and more than half were Saudi nationals. The mean experience of the participants was (6.3±3.7) years. The nurses at the emergency department had satisfactory knowledge about most of the core emergency nursing competencies with some areas such as genitourinary, gynecological assessment, and documentation being deficient. Increasing age, being non-Saudi, married and nurse supervisor were associated with higher competencies.

Conclusions

Nurses generally had satisfactory knowledge of most of the core competencies of nurses in the emergency department in the Qassim region. However, there were deficiencies in core competencies in some domains. There is a need to provide on-the-job training and coaching for emergency nurses to improve their competencies and the quality of emergency care in the hospitals.

## Introduction

Today's healthcare environment is very complex and presents a significant challenge for nurses. In particular, emergency situations require nurses to resort to interconnected and complex issues, such as the connection between patient management and urgent decisions in uncertain circumstances [1].

There is a close relationship between the clinical competence of nurses and the quality of care [2]. Lack of knowledge and the variation in the professional competencies in different skills such as critical thinking and research aptitude, clinical care, leadership, legal and ethical practices, professional development, interpersonal relationships can lead to lowering the quality of healthcare services to care recipients [3]. There is a lack of high-quality studies exploring the clinical competence of nurses in critical areas such as emergency department.

The nature of emergency nursing practice requires appropriate guidance and practice to address a wide range of patients, conditions, supplies, equipment, procedures, policies and processes. Nurses working in such stressful situations need to have the ability to quickly assess and analyze the patient's problem with a systematic and professional approach and find solutions that prioritize urgent interventions.

In the context of constant changes in medical technology and the roles and responsibilities of nurses, it is important that not only nurses maintain and develop their clinical competencies, but also that nurses and managers evaluate these competencies to ensure the quality and safety of the attention [4]. There is a close relationship between nurses' clinical competency and quality of care. However, there is a lack of high-quality studies exploring the basic competencies that should be mastered by the emergency nurses and the nurses' clinical competency criteria in the emergency departments, especially in Saudi Arabia [5]. Nurses are important members of the healthcare system, and their clinical competence is essential, especially in the emergency room. In 2019, among the total health workforce in the Ministry of Health, 45.3% were nurses [6].

Nursing as a clinical discipline is evolving day by day and nurses are involved as a key member in various settings, including emergency rooms. Nurses must maintain their professional competence and improve the health system they need to continually assess and prioritize their indicators of clinical competence. Assessment of clinical competence is difficult due to the lack of clear standards. The Emergency Nurses Association defines competence as “the habitual and judicious use of knowledge, technical skills, communication, clinical reasoning, emotions, values, and reflection in daily practice of the emergency nurses for the benefit of the individual and community being served” [7].

The Theory of the Structure of Nursing Knowledge (Nursing Knowledge Pyramid) can be utilized as a theoretical framework for the current research [8]. A theory of the structure of nursing knowledge is proposed. Using retrospective reasoning to build upon an existing theory. The goal of the Nursing Knowledge Pyramid is to integrate disparate forms of nursing knowledge into a comprehensive, coherent, and useful structure to enhance the learning, development, automation, and accessibility of nursing knowledge.

Given the scope of the roles of nurses in different clinical settings, it is necessary to develop specific tools to assess the clinical competence of nurses [9]. These competencies can then be used as clinical guides, either in the assessment or in the training and preparation of nurse practitioners in clinical settings such as emergency rooms [10]. The literature on nursing competencies in Saudi Arabia is scarce and available studies are focused on nurses in general rather than emergency care [3] or expatriate nurses [11]. Therefore, the aim of the study was to assess the core emergency nursing competencies of nurses working in emergency department of hospitals in Qassim Saudi Arabia. The secondary objective was to explore the determinants of competencies among nurses. The results of this research can be used to suggest recommendations for constructing a tailored instructional program for the emergency nurses at Saudi hospitals aiming to improve their knowledge regarding the core nursing competency, based on the needs assessment, in order to improve nurses practice and assure safe nursing care delivery at the emergency departments.

## Materials and methods

Study design and setting

A cross-sectional study was conducted among nurses working in the emergency department in different hospitals in the Qassim region. Qassim region is located in the central part of Saudi Arabia and has an estimated population of about 1.4 million people. There are 19 hospitals under the Ministry of Health (MoH) in the region with a bed capacity of around 2900. Out of these, one hospital is dedicated for psychiatric illnesses and one for long-term care. As of 2021, there is a total of 5788 nurses, including midwives, working in MoH hospitals in the Qassim region [6].

Sample size

The sample size was calculated using OpenEpi Software (Version 3.01, Copyright (c) 2003, 2008 Andrew G. Dean and Kevin M. Sullivan, Atlanta, GA, USA). Since the target population was finite (N= 420 nurses), we used finite population correction while estimating the sample size. At a 95% confidence level, with a 5% bound on error, and a 50% anticipated frequency, the required sample was 201.

Sampling

Convenient sampling was used to recruit participants from the emergency departments of seven public sector hospitals in Qassim, which included King Fahad Specialist Hospital, Buraidah Central Hospital, Maternal and Children Hospital, Bukayriyah General Hospital, King Saud Hospital, Ar Rass General Hospital, and Al Midhnab General Hospital. These hospitals were selected because they are located in the largest cities of the Qassim region with more than 80% of the population living in these cities. 

First, we obtained administrative approval to conduct the research in the respective hospitals. Then we approached the nursing departments of each of the study sites and explained the purpose and procedure of the study to the representatives of nursing departments and requested to share the questionnaire link with the nurses working in the emergency department of their hospitals. 

Data collection instrument and procedure

The current study utilized a standardized tool that encompasses a sociodemographic datasheet and a self-administered questionnaire which has nine main items or themes, along with sub-items asking about the proficiency of the nurse concerning some basic and core competencies at the emergency department from their own perception and using a five-point Likert scale ranging from not applicable (0), developmental (1), Competent (2), Desire to enhance (3), and excellent (4) [12]. As the study targeting the nurses working at Hospital in Qassim, the questionnaire was used in its original English language without any translation. Piloting of the questionnaire was carried out prior to conducting the data collection stage of the research to assure the clarity and feasibility of the questionnaire before being submitted to participants. Finally, the tool was found to be clear and easy to be filled up in 10 minutes. It was used without conducting major modifications of the questionnaire, however, the sample collected from the 20 participants of the piloting was not included in the final results and was excluded from the study sample.

The electronic questionnaire was distributed using social media for all nurses working in the emergency departments of selected hospitals in the Qassim region. The first page of the questionnaire contained information about the research objectives and informed consent.

Data analysis

Data was collected and analyzed utilizing the Statistical Package for Social Sciences (SPSS version 26, IBM Corp., Armonk, USA)). A descriptive analysis of data was done through the use of the mean and standard deviation. However, the data related to the core competencies of the nurses working in the emergency department were represented in tables utilizing descriptive statistics using frequencies and numbers. For calculating the overall mean score of competencies, we excluded ‘not applicable’ from the Likert scale and calculations, and interpretations were made on a scale of 1-4. Univariate and multivariate regression analyses were carried out to identify the determinants of competencies. Variables in the final model were retained based on their contribution in the model as assessed by statistical significance and R2 values. Multicollinearity among the potentially relevant variables was assessed. A strong correlation was observed between age and years of experience, therefore, only age was retained in the final model. Adjusted beta (B) along with the associated 95% confidence interval (CI) were determined.

Ethical issues

Ethical approval was obtained from Qassim Regional Research Ethics Committee (Approval number: 1443-529567). The confidentiality of the participants was ensured and no data on personal identifiers was collected.

## Results

A total of 213 nurses participated in the study. The mean age of the participants was 32.9±4.6 years. Almost two-thirds of them were females (69.5%). More than half were Saudi nationals (58.5%) and a similar proportion held a bachelor's degree in nursing (58.7%). For the job title, almost two-thirds of the participants were nurse specialists (65.3%), while the fewest were nurse supervisors (6.1%). The mean experience of the participants was 6.3±3.7 years (Table 1).

**Table 1:** Sociodemographic and professional characteristics of nurses working in hospitals of Qassim, KSA KSA: Kingdom of Saudi Arabia

Variable	% (n)
Age	
Mean (SD)	32.9 (4.64)
Gender	
Female	69.5 (148)
Male	30.5 (65)
Nationality	
Saudi	58.5 (124)
Non-Saudi	41.5 (88)
Educational level	
Diploma	32.4 (69)
Bachelor	58.7 (125)
Master	6.6 (14)
Ph.D.	2.3 (5)
Marital Status	
Single	40.8 (87)
Married	48.4 (103)
Divorced	8.0 (17)
Widow	2.8 (6)
Work experience Mean (SD)	6.3 (3.66)
Workplace	
King Fahad Specialist Hospital	19.2 (41)
Buraidah Central Hospital	17.4 (37)
Buraidah Maternal and Child Hospital	26.8 (57)
Bukayriah General Hospital	10.3 (22)
King Saud Hospital	14.1 (30)
Al Ras General Hospital	7.5 (16)
Al Midhneb Hospital	4.7 (10)
Job title	
Nurse assistant	10.8 (23)
Nurse Specialist	65.3 (139)
Charge Nurse	9.9 (21)
Head Nurse	8.0 (17)
Supervisor	6.1 (13)

Table 2 presents the self-reported level of clinical competencies of emergency nurses at the governmental hospitals in the Qassim region. Less than half of the nurses (40%) reported that they were excellent in demonstrating knowledge and ability to apply critical thinking and clinical judgment to adapt health assessment to emergency nursing. In the domain of neurological assessment, the highest proportion of the excellent rating was in oxygen monitoring (51.2%) followed by assessment of consciousness (46.5%). The weakest areas of competencies in this domain were pupil reaction (17.5%) and sensory functions (14.6%). Thirty-eight percent of the respondents rated themselves excellent in peripheral assessment while about 41% rated excellent in the assessment of color, sensation, movement (CSM)/color, warmth, circulation, movement (CWCM). In respiratory assessment, about 53% and 47% rated themselves as excellent in the assessment of cyanosis, and respiratory rate, rhythm and quality, respectively. The highest proportion of the developmental level of competency was rated in the assessment of tracheal tug (27%) and jugular vein distension (25.5%). 

**Table 2:** Emergency nursing core competencies’ self-rating by nursing staff in emergency departments in Qassim, KSA KSA: Kingdom of Saudi Arabia

Systems' assessment competencies	Not applicable	Developmental	Competent	Desire to enhance	Excellent
Details of the assessment competencies	% (n)	% (n)	% (n)	% (n)	% (n)
Critical thinking and clinical judgment					
Demonstrate knowledge and ability to apply critical thinking and clinical judgment to adapt health assessment to emergency nursing	6.7 (14)	9.1 (19)	16.3 (34)	27.9 (58)	39.9 (83)
Neurological assessment					
Neurological vital signs	6.1 (13)	13.7 (29)	13.7 (29)	32.5 (69)	34 (72)
Generalized seizure activity	5.7 (12)	12.7 (27)	18.9 (40)	24.5 (52)	38.2 (81)
Glasgow coma scale	6.1 (13)	13.1 (28)	14.1 (30)	30.5 (65)	36.2 (77)
Level of consciousness	3.8 (8)	14.1 (30)	12.2 (26)	23.5 (50)	46.5 (99)
Loss of consciousness	2.8 (6)	11.8 (25)	15.2 (32)	23.2 (49)	46.9 (99)
Motor function	2.3 (5)	14.1 (30)	16.9 (36)	25.4 (54)	41.3 (88)
Pupil reaction	6.6 (14)	17.5 (37)	19.8 (42)	26.4 (56)	29.7 (63)
Oxygen saturation	2.4 (5)	11.4 (24)	10.9 (23)	24.2 (51)	51.2 (108)
Sensory function	2.8 (6)	14.6 (31)	14.2 (30)	28.3 (60)	40.1 (85)
Visual acuity	3.3 (7)	11.8 (25)	23.1 (49)	26.9 (57)	34.9 (74)
Peripheral neurovascular assessment					
Peripheral assessment	5.2 (11)	13.1 (28)	19.2 (41)	24.4 (52)	38 (81)
Color, sensation, movement (CSM) / color, warmth, circulation, movement (CWCM)	4.2 (9)	16 (34)	20.2 (43)	18.8 (40)	40.8 (87)
Respiratory assessment					
Accessory muscle use	6.6 (14)	11.7 (25)	16.9 (36)	31.5 (67)	33.3 (71)
Auscultation of lung fields	4.7 (10)	18.9 (40)	22.6 (48)	25 (53)	28.8 (61)
Color	3.3 (7)	11.7 (25)	14.6 (31)	30.5 (65)	39.9 (85)
Cough	3.3 (7)	9 (19)	16 (34)	27.8 (59)	43.9 (93)
Cyanosis	2.8 (6)	8.9 (19)	16 (34)	19.7 (42)	52.6 (112)
Expectorated secretions	5.2 (11)	10.9 (23)	17.1 (36)	25.6 (54)	41.2 (87)
Jugular vein distention	8.5 (18)	25.5 (54)	24.1 (51)	25.5 (54)	16.5 (35)
Oxygen saturation per monitor	3.3 (7)	12.3 (26)	18 (38)	24.2 (51)	42.2 (89)
Peak flow	4.2 (9)	12.3 (26)	19.3 (41)	33 (70)	31.1 (66)
Respiration – rate, rhythm, quality	2.8 (6)	13.1 (28)	15.5 (33)	22.1 (47)	46.5 (99)
Tracheal deviation	11.7 (25)	22.1 (47)	26.8 (57)	22.1 (47)	17.4 (37)
Tracheal tug	11.7 (25)	27.2 (58)	24.4 (52)	22.1 (47)	14.6 (31)
Cardiovascular assessment					
Application of ECG leads and monitoring	3.8 (8)	9.9 (21)	18.8 (40)	23.5 (50)	44.1 (94)
Application of external cardiac monitoring leads	2.8 (6)	10.8 (23)	20.7 (44)	18.8 (40)	46.9 (100)
Auscultation of chest	4.2 (9)	17.8 (38)	25.4 (54)	25.4 (54)	27.2 (58)
Blood pressure, postural, and palpable BP	1.9 (4)	11.7 (25)	20.2 (43)	24.9 (53)	41.3 (88)
Heart rate, rhythm, and peripheral pulses	2.3 (5)	12.2 (26)	16.9 (36)	22.5 (48)	46 (98)
Heart sounds (S1,S2)	8.9 (19)	21.1 (45)	28.6 (61)	29.6 (63)	11.7 (25)
Capillary refill	4.2 (9)	13.6 (29)	20.7 (44)	25.8 (55)	35.7 (76)
Perspiration	5.7 (12)	15.6 (33)	21.2 (45)	25.9 (55)	31.6 (67)
Pitting edema	2.9 (6)	13.4 (28)	15.8 (33)	21.5 (45)	46.4 (97)
Signs or symptoms of infection	2.8 (6)	15.2 (32)	14.2 (30)	28.9 (61)	38.9 (82)
Musculoskeletal assessment					
Soft tissue injuries / multiple system injuries / trauma	4.7 (10)	9.9 (21)	14.6 (31)	38.5 (82)	32.4 (69)
Fractures	3.8 (8)	11.7 (25)	15 (32)	30.5 (65)	39 (83)
Dislocations	5.6 (12)	12.2 (26)	21.1 (45)	29.1 (62)	31.9 (68)
Degenerative disorders	5.2 (11)	18.4 (39)	20.3 (43)	27.8 (59)	28.3 (60)
Mobility and ambulation	4.7 (10)	14.2 (30)	20.3 (43)	30.2 (64)	30.7 (65)
Genitourinary assessment					
Assess patency of urinary catheter	5.6 (12)	12.7 (27)	18.8 (40)	29.6 (63)	33.3 (71)
Bladder scanning	7 (15)	17.4 (37)	23.5 (50)	29.6 (63)	22.5 (48)
Check external genitalia for signs of trauma	6.1 (13)	14.6 (31)	17.4 (37)	33.8 (72)	28.2 (60)
Insert and remove catheter	17.8 (38)	15 (32)	20.7 (44)	23.9 (51)	22.5 (48)
Lab findings	5.2 (11)	18.3 (39)	25.4 (54)	27.7 (59)	23.5 (50)
Monitor urinary output	4.7 (10)	11.3 (24)	17.4 (37)	24.9 (53)	41.8 (89)
Palpate for bladder distension	6.1 (13)	19.3 (41)	20.8 (44)	27.8 (59)	25.9 (55)
Strain urine for calculi	9.5 (20)	24.6 (52)	23.2 (49)	24.6 (52)	18 (38)
Gynecological assessment					
Gravida, para, abortions	31.5 (67)	13.6 (29)	17.4 (37)	18.8 (40)	18.8 (40)
Last normal menstrual period (LNMP)	30 (64)	13.6 (29)	19.2 (41)	19.2 (41)	17.8 (38)
Vaginal discharge or blood loss	30.5 (65)	15 (32)	15 (32)	18.8 (40)	20.7 (44)
Previous gynecological surgery	30.5 (65)	13.1 (28)	20.2 (43)	15 (32)	21.1 (45)
Cramping, labor pains	32.4 (69)	13.6 (29)	10.8 (23)	23 (49)	20.2 (43)
Length of last labor	31.5 (67)	12.7 (27)	16 (34)	19.2 (41)	20.7 (44)
Previous premature births	31.1 (66)	13.7 (29)	11.3 (24)	21.7 (46)	22.2 (47)
Birth control history	32.1 (68)	12.3 (26)	13.7 (29)	18.4 (39)	23.6 (50)
Pregnancy testing	32.1 (68)	12.7 (27)	12.7 (27)	17.5 (37)	25 (53)
Documentation					
Demonstrate the knowledge and ability to document and report assessment findings to the appropriate health professional.	32.5 (69)	14.6 (31)	12.3 (26)	17.5 (37)	23.1 (49)

In the domain of cardiovascular assessment, a high level of competency was reported in the application of external cardiac monitoring leads (47%), pitting edema (46%), and heart rate and rhythm (46%). Poor competencies were in heart sounds (21%), and auscultation of the chest (18%).

Palpation for bladder distension was the competency with the highest excellent rating (42%) while for most other assessments in the genitourinary system, the excellent rating was generally below 25%. Similar were levels of competence in the gynaecological assessments where only pregnancy testing was rated excellent by only 25% of the participants while the rest of the assessments were around 20%. In the domain of documentation, only 23% rated themselves excellent, while 32.5% mentioned it as not applicable to their job.

Multivariate linear regression analysis showed that age was positively associated with the overall score of competencies adjusted B 0.059 (95% CI: 0.031:0.087). Non-Saudi nurses showed higher competency adjusted B 0.503 (95% CI: 0.265:0.741). Being ever married was associated with a lower overall competency score adjusted B -0.329 (95% CI: -0.593:-0.064). Nurse supervisors had higher competency as compared to nurse assistants adjusted B (95% CI: 0.032:1.141). There was no significant association of academic qualification with overall competencies (Table 3).

**Table 3:** Factors affecting core emergency competencies of nursing staff in Qassim, KSA ^¥^ Variables were mutually adjusted for each other in multivariate linear regression model. KSA: Kingdom of Saudi Arabia

Variable	Adjusted^¥^ B (95% CI)	p-value
Age	0.059 (0.031:0.087)	0.000
Gender		
Female	Ref	
Male	0.213 (-0.039:0.465)	0.097
Nationality		
Saudi		
Non-Saudi	0.503 (0.265:0.741)	0.000
Marital status		
Never married	Ref	
Ever married	-0.329 (-0.593:-0.064)	0.015
Qualification		
Nursing diploma	Ref	
Bachelors	-0.129 (-0.373:0.115)	0.299
Master	-0.11 (-0.543:0.323)	0.618
Job position		
Nurse assistant	Ref	
Nurse Specialist	0.239 (-0.127:0.605)	0.200
Charge Nurse	0.124 (-0.368:0.617)	0.619
Head Nurse	0.245 (-0.284:0.773)	0.362
Supervisor	0.586 (0.032:1.141)	0.038

## Discussion

Our study is among a few from Saudi Arabia to assess the competencies of nurses in Saudi Arabia. This study highlighted self-rated core competencies among emergency nurses at the public hospitals in the Qassim region, within the domains of systems' assessment competencies such as critical thinking and clinical judgment, clinical assessments, and documentation of the assessment. The clinical assessments included neurological, peripheral neurovascular, respiratory, cardiovascular, musculoskeletal, genitourinary, and gynecological assessments. Overall, the majority of nurses have reported core competencies in mostly all the domains in “excellent” categories as compared to other categories such as “developmental, competent, and desire to enhance”. However, analyzing individual competencies showed that at least 40% of nurses reported excellent competency in critical thinking and clinical judgment for demonstrating knowledge and health assessment. For neurological assessment, oxygen monitoring (51.2%), and consciousness assessment (46.5%) received excellent scores. While nurses reported themselves as excellent in respiratory rate (53%) and cyanosis (47%) assessment. For cardiovascular domain assessment, excellent competency was reported in the application of external cardiac monitoring leads (47%), pitting edema (46%), and heart rate and rhythm (46%). However, for gynecological assessment, only 25% of nurses reported excellent competency in pregnancy testing, while for overall documentation only 23% rated themselves as excellent.

Studies from the United States and Iran to assess the clinical competencies of emergency nurses showed that the overall competency of the emergency nurses indicated a good level of perceived competence [13, 14]. These findings are similar to our results where there was a good level of competence reported by emergency nurses.

Similar to our findings, a study among nurses in Saudi Arabia reported a lower level of competence with respect to critical thinking [3]. Critical thinking among nursing professionals especially in emergency care plays a very crucial role in decision-making for patient management and clinical judgment in a critical situation. A recent study showed a positive significant correlation between the critical thinking of emergency care nurses and their clinical decision-making [15].

Other researchers have explored that the higher self-reported competency levels have been associated significantly with frequent clinical skills performance in the respective domains [3, 14]. McCarthy et al. reported that the nurses in emergency departments frequently performed diagnostic functions in the clinical skills domain and helping roles and found a higher perceived level of competency for these skills [7]. On the other hand, researchers identified the association of less frequently demonstrated clinical skills with advanced and specialized nursing care [16]. Therefore, it is crucial for nurses in emergency care to acquire more expertise and training in order to gain more experience on less-performed clinical tasks and achieve a higher level of competency. As a result, failures and errors can be reduced due to incompetency to detect patients’ status correctly and the management [17]. Nevertheless, advanced nursing roles and practices have been expanding worldwide, but confusion in role clarity, different cultures of nurses, authority and unclear chain of command status lead to worse performance of certain skills by nursing staff [18, 19]. According to the Emergency Nurses Association, emergency nurses are required to take various additional courses to develop their skills and use these skills to assess, plan, intervene, and evaluate patients in the emergency department [20].

Documentation and reporting about patient management especially in emergency care by nurses is an important competency; however, research has highlighted that the quality of the nursing reporting system is quite far from standard and needs improvement [21]. This research finding is comparable to our study result which reported 25% of nurses were showing excellent documentation skills. Researchers have also explored the barriers of sub-standard reporting system by emergency nursing staff which includes documentation competency itself, work burden and burnout, poor coordination and perceived control of the system [22, 23]. Hence, a higher level of skills with respect to the nursing assessment followed by comprehensive, accurate, and concise documentation is much needed, and healthcare organizational support to the nursing staff especially in the emergency setting is vital for the fulfilment of the documentation competency so that they should not avoid it due to heavy workload [24].

In our study, the age of nursing staff has been positively associated with overall competencies score. Another research with some similar findings has reported that age has a significant impact on critical thinking and critical judgment [15].

Another determinant has been found as non-Saudi nurses associated with higher clinical competency in our study. Literature supports the findings as cultural differences have a significant impact on higher clinical competence. This could be due to the fact that they are nursing staff from countries other than the Saudi regions make extra efforts to prove their competency levels at their best [19].

In our study, gender was found to be an insignificant determinant of clinical competency among emergency care nurses. A similar finding from a cross-sectional study conducted to evaluate clinical competence among critical care nurses in Iran reported that gender has no impact on clinical competency [25]. On the other hand, another cross-sectional study was carried out to assess the effect of critical thinking on clinical judgment among critical care nurses and found that gender has a significant impact on critical thinking and clinical decision-making [15].

Nurse supervisor has shown higher competency as compared to nursing assistant in our study, which means specialized nurses have an important role to play in professional clinical skills competencies. This could be due to more years of experience and higher qualifications. However, in our study, academic qualification has not been found to be associated with clinical competence. Research suggests that working experience significantly impacts the clinical decision-making process, especially in the emergency department as nurses' reflexes to identify clinical cases get refined due to long work years of exposure facing various types of clinical cases [3, 15]. Additionally, a recent study explored the clinical competence adequacy in newly graduated nurses in Sweden, and they found that there was a shortage of experience in recently graduated nurses, and they were struggling in developing professional competence [15, 26]. Reflection on teaching and learning is an important component in professional skills competence development and the transition from teaching to practical clinical skills demonstration is a challenging yet crucial part, especially in critical care set-ups such as emergency care. Therefore, this gap between theoretical knowledge and practice should be fulfilled [27]. In addition, the researchers identified that the leadership role of nurses matters significantly in managing the nursing staff in emergency care. The behaviour and positive attitude of senior nurse managers, with transformational leadership skills, have been proven to affect the subordinates’ commitment towards learning [22].

Our study has some strengths in identifying the level of competencies in the clinical and administrative domains in detail among nursing staff working in emergency departments. This information can help in devising targeted strategies for the competencies domain that need more attention. The study was conducted in the emergency departments of seven public hospitals with large geographic coverage. The sample size in our study was scientifically calculated to capture a sufficient study population. These factors make our sample largely representative of the emergency nurses' population in the region. The standardized tool comprising nine comprehensive domains having the criteria of the Likert scale was used for the response collection. A few limitations can be summarized as well for our study. The convenient recruitment of study participants could have introduced a selection bias which may affect the generalizability of our findings. However, we assume this to be of little significance as we covered a large sample from the largest hospitals in the region. The responses from the participants were self-reported which could be subjected to response bias. Additionally, an information bias could be present due to the carrying out of electronic data collection. There is also the possibility of social desirability bias in responses as respondents might rate themselves at higher levels of competence. Additional investigation of the clinical competence of nurses using direct observation of their performance and later correlation of the performance with the self-reported level of competency is needed.

## Conclusions

The nurses in the emergency department have reported satisfactory knowledge about most of the core emergency nursing competencies in our study. However, there is still a segment of nurses who are not up to the mark and reported some deficiencies in their perceived competencies. This calls for more frequent training opportunities for the nursing staff by educators and senior nursing specialists, which should be provided. Targeted strategies in specific clinical skills domains can be devised for the training and upgradation of skills among emergency care nurses, which will be time and resources efficient.
